# A streamlined set-up for Lloyd’s mirror interference lithography, using a single-mode-fibre-coupled laser

**DOI:** 10.1038/s41378-026-01186-4

**Published:** 2026-06-10

**Authors:** Enkui Lian, Eleni Perivolari, Yu Liu, John C. deMello

**Affiliations:** 1 Department of Chemistry and Bioengineering, Norwegian University of Science and Technology, Trondheim, Trøndelag Norway; 2 Department of Materials Science and Engineering, Norwegian University of Science and Technology, Trondheim, Trøndelag Norway

**Keywords:** Optical materials and structures, Nanophotonics and plasmonics

## Abstract

We describe a streamlined set-up for Lloyd’s-mirror-based laser interference lithography (LM-LIL), using only a fibre-coupled single-frequency laser (SFL), a plane mirror and an optional half-wave plate as the optical components. Using an SFL with a factory-set, pigtailed single mode fibre as the LM-LIL light-source greatly simplifies experimental implementation by removing the usual need to manually align a free-space laser-beam to a pinhole-based spatial filter or external fibre. The resulting system is inexpensive, robust and easy to use, allowing for the straightforward preparation of one- and two-dimensional arrays with pitches down to the sub-220-nm level when using a 405-nm laser. Full details of the experimental set-up are provided, together with a step-by-step description of sample preparation, exposure and subsequent processing.

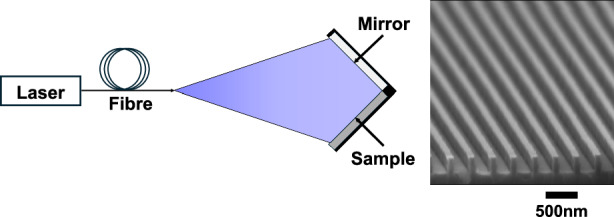

## Introduction

Laser interference lithography (LIL) uses overlapping beams of coherent light to transfer a high-resolution interference pattern to a photoresist^1^. Visible-light LIL allows for the direct fabrication of highly ordered one- and two-dimensional arrays of lines and spots in the exposed photoresist, with a pitch down to a few hundred nanometres. The pattern may then be transferred to a variety of other materials by standard lithographic processes such as lift-off or etching. LIL enables the rapid fabrication of highly ordered arrays spanning typical areas of several square centimetres, and can be used to make a wide variety of optically, electronically and chemically functional surfaces.

In photonics, key applications of LIL include the fabrication of diffraction gratings^2^, nano-gratings^3^, photonic crystals^4^ and distributed feedback lasers^5^, as well as plasmonic^6^ and metamaterial^7^ arrays for manipulating light at the nanoscale. In electronics, it is used to create periodic nanostructures of nanowires^8,9^, quantum dots^10^ and magnetic thin films^11^ for optoelectronic and data storage applications. In biology, it is used to create cell culture scaffolds^12^, biosensors^13^ and patterned surfaces for tissue engineering^14^. It is also commonly used for surface modification to create surfaces of defined hydrophobicity or friction^15,16^.

While LIL is a straightforward technique, there are many potential traps and pitfalls that can prevent a high-quality pattern from being obtained. Considerable care is needed in many aspects of its implementation to ensure success. Unfortunately, detailed descriptions of the practical aspects of LIL are hard to find, with most LIL-related articles focusing either on tweaks to the experimental set-up or on the final patterned structures. The purpose of this paper is to provide a detailed account of the practical implementation of LIL, using a highly streamlined LIL system based on relatively inexpensive, modern hardware. The system is designed to operate in a small room with electrical power sockets, but no special vibration-management, air-handling or climate-control facilities (although ideally it should be housed in a cleanroom that *does* provide such facilities).

**Fig. 1:**
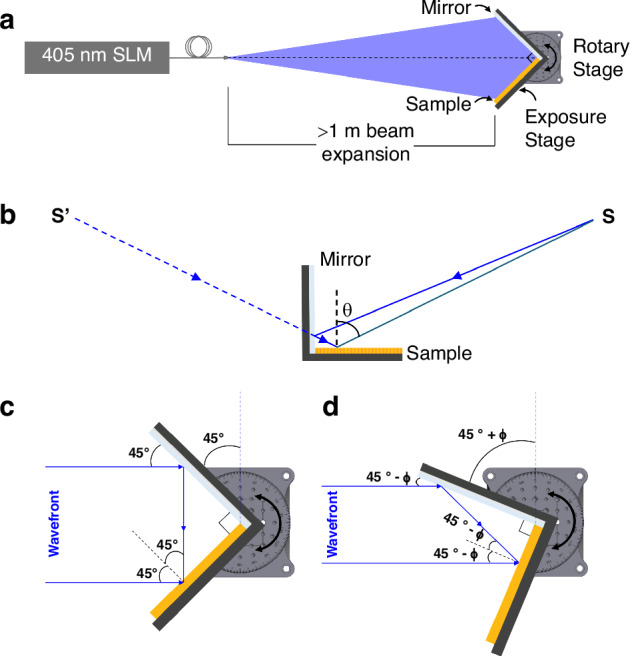
Schematic showing geometry used for Lloyd's mirror interference lithography. **a** Set-up for LM-LIL, using a 405 nm fibre-coupled single frequency laser, operating on a single longitudinal mode (SLM). The divergent beam from the laser’s single mode fibre (SMF) is allowed to expand over a typical distance of 1–3 m. Part of the expanded beam falls on a rotatable exposure stage where a sample and a mirror are held at 90° to one another. Part of the beam falls directly on the sample and another part of the beam strikes the sample after reflection from the mirror. Overlap of the direct beam and the reflected beam generates a fringe pattern. (Note, although the laser beam is shown only just filling the exposure stage, in reality the beam is much wider, and the exposure stage sees only a small fraction of the full beam). **b** Schematic showing the equivalence between Lloyd’s-mirror-LIL and multiple-beam LIL, with the reflected beam in LM-LIL effectively originating from the virtual image point **S**’. The angle $\theta$ varies across the substrate, introducing a position-dependence to the pitch that can be minimised by placing the source S far from the sample. **c** Schematic showing a direct light-ray and a reflected light-ray meeting at a point on the sample when the exposure stage is far from the source and in its home position. The direct and reflected rays strike the sample at angles of ${{\pm}{45}^{\circ}}$ with respect to the sample normal. **d** Schematic showing a direct light-ray and a reflected light-ray meeting at a point on the sample when the exposure stage has been rotated anticlockwise through an angle $\phi$. The direct and reflected rays strike the sample at reduced angles of ${{\pm}({45}-{\phi})^{\circ}}$ with respect to the sample normal, causing an increase in the pitch of the interference pattern, see Eq. (1)

Our set-up uses the simplest form of LIL known as the Lloyd’s mirror configuration^17^. Lloyd’s mirror LIL (LM-LIL) requires only a laser, some simple optics to generate a spatially filtered wavefront, and a mirror to form a reflected wavefront that interferes with directly incident light on the sample, generating a series of parallel interference fringes (see e.g. Fig. 1a). A single exposure generates a 1D array of lines (after processing of the photoresist), while sequential exposures at different substrate orientations can be used to generate two-dimensional arrays of circular or elliptical spots on a square or hexagonal lattice^18,19^. The Lloyd’s mirror approach is distinguished by its excellent resilience to vibration since the mirror and sample can be mounted on the same rigid supporting mount, meaning any mechanical disturbance will, to first order, affect them equally and hence will not disturb the optical path difference and the resulting interference pattern. In addition, LM-LIL shows moderately good resilience to air-turbulence since the direct and indirect beams follow similar paths from the fibre-tip to the sample.

In an alternative approach to interference lithography—multiple-beam LIL (MB-LIL)—two or more coherent beams derived from the same source overlap to generate the interference pattern^20^. The connection between LM-LIL and MB-LIL is evident from Fig. 1b, which shows that the reflected beam in LM-LIL can be considered as arising from a virtual point-source **S’** located at the image point of the real source **S**. In practice, MB-LIL offers greater flexibility than LM-LIL as the phases, polarisations and amplitudes of the individual beams can be varied with respect to one another, generating a wide variety of 2D fringe-patterns. On the other hand, MB-LIL is much more sensitive to the disruptive effects of vibrations and air-flow because the beams follow very different paths through space and therefore experience uncorrelated disturbances that cause drift in their relative phases. Dynamic compensation for the phase drift (‘fringe locking’) is therefore essential to achieve a stable interference pattern^19,21^, which adds considerable cost and complexity to the system. Most ‘entry-level’ LIL systems therefore exploit the Lloyd’s mirror approach. A further advantage of MB-LIL over conventional LM-LIL is that 2D patterns can be generated in a single exposure without needing to carry out multiple exposures at different sample orientations, although multi-mirror variants of LM-LIL also allow for this^22–24^.

Typical implementations of LM-LIL use a free-space laser in conjunction with a spatial filter^19,22,25^ to produce a clean divergent beam that is free from aberrations due to dust, dirt and scratches in the optics. Without spatial filtering, the interference pattern used to expose the sample is contaminated by spatial noise that distorts the fringe-pattern and leads to numerous defects in the final array. Conventionally, spatial filtering is achieved by using a microscope objective or an aspheric lens to focus the laser beam onto a narrow pinhole^26^. Light at the centre of the focused spot corresponds to the wanted planewave component of the incoming laser beam, while light away from the centre is due to off-axis components of the light (noise). Hence, by aligning the pinhole to the centre of the focused spot, the clean part of the input beam can be passed while higher frequency spatial noise is blocked.

Although a lens-pinhole spatial filter (LPSF) is certainly capable of generating a clean divergent beam suitable for LIL, it has some practical drawbacks. In particular, since the pinhole must be located at the centre of the focused beam, any wander of the laser beam will cause the fringe pattern on the photoresist to drift uncontrollably during the course of the exposure^27,28^, resulting in blurring (or even loss) of the final pattern. A high-quality laser with good pointing-stability is therefore needed to achieve good results, adding cost to the set-up. In addition, pinholes are generally sold in a rather limited range of sizes, and finding one that works well can be challenging: the pinhole must be small enough to block all of the spatial noise, but not so small that it diffracts the incoming beam and so distorts the transmitted wavefront. Typically, it is necessary to oversize the pinhole to avoid diffraction at the expense of some unwanted noise components leaking through.

To get around the problems of LPSF, a few papers have reported the use of a single-mode optical fibre as a spatial filter^27–29^. Optical fibres support a finite number of ‘guided modes’ —travelling-wave solutions to Maxwell’s equations—that propagate along the core of the fibre with minimal attenuation and unchanging transverse electric field distributions^30^. The fundamental guided mode (LP₀₁) is a featureless circularly symmetric wave with a Gaussian-like variation in electric field strength in the transverse direction (Fig. S1a), whereas higher order modes have more complex electric field distributions with multiple lobes (see e.g. Fig. S1b, c). In a single mode fibre (SMF) the core radius $a$ is so small that only the LP₀₁ mode can propagate through it, with energy from all other modes—including spatial noise from the input beam—being dissipated in the cladding and its surrounding jacket. As a result, the light emitted by an SMF is a featureless, linearly polarised, Gaussian-like, divergent beam that is derived purely from the LP_01_ mode and therefore unaffected by beam wander. (Variations in beam-angle affect only the efficiency with which light is coupled into the fibre, with the output power averaging-out to a constant value over typical exposure times). The divergence of the output beam from an SMF is determined by the numerical aperture of the fibre, which is typically in the range 0.1–0.15.

For distances substantially greater than $\pi {a}^{2}/\lambda$ —typically some tens of microns for a visible-light SMF—the wavefront closely approximates part of a spherical wave^31^, with a Gaussian-like variation in electric field strength in the transverse direction. To achieve linear fringes of uniform amplitude, the light emitted from the fibre tip should resemble a plane wave, i.e. the wavefront should be both planar and uniform in intensity over the area of the sample. This can be achieved by placing the exposure stage far from the fibre-tip and in line with the beam-axis so that the stage is illuminated by only a small, central portion of the expanding beam (across which the wavefront is approximately flat and the electric field strength is approximately constant). Any non-planarity in the interfering wavefronts at the sample leads to hyperbolic distortion (non-linearity) of the fringe pattern^32^, while variations in the electric field strength across the sample lead to fringes of varying amplitude. (Referring to Fig. 1b, it is apparent that one consequence of having spherical wavefronts is that the angle $\theta$ at which the direct and reflected beams strike the sample depends on the position within the sample. Closer to S, i.e. to the right of the sample, the beams strike the sample at smaller angles to the normal, resulting in a larger fringe spacing, see Eq. 1 below).

Previous reports of SMF-based LIL have used an SMF coupler to launch a free-space laser beam into the fibre, which adds significant inconvenience and cost to the experimental set-up. Further, since the core of an SMF is typically less than 10 μm in diameter, coupling losses can lead to a substantial reduction in intensity relative to the input beam. Here, we instead use the direct output from a fibre-coupled laser as our LIL light-source. By using a pigtailed laser^33^, in which the SMF is optimally and permanently aligned to the laser facet during manufacturing, all manual alignment issues are removed from the set-up, substantially simplifying implementation. Beyond the fibre-coupled laser itself, the only additional optics needed for a complete LM-LIL set-up are a plane mirror and an optional half-wave plate. The resulting system is about the simplest set-up conceivable for carrying out high quality LM-LIL.

## Experimental procedure

### Experimental set-up

**Fig. 2:**
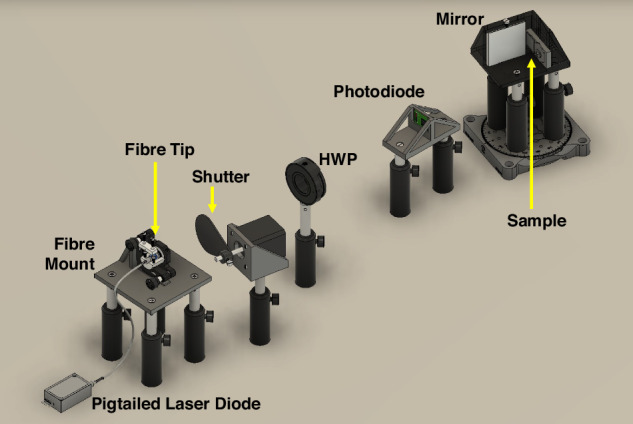
Schematic of complete set-up (not to scale). The light-source is a 20-mW fibre-coupled single frequency laser diode that emits at 405 nm. The fibre is a single-mode fibre (S405-XP), terminated with a coreless end-cap to minimise the risk of burnt debris collecting on the fibre tip. The fibre-tip is held in a 3D-printed mount supported by four optical posts for stability. Just after the fibre tip is a mechanical shutter driven by a stepper motor, followed by an optional half-wave plate (HWP), which is used to set the polarisation axis of the laser light to vertical. At the far end of the set-up, a 3D-printed exposure stage holds the mirror and the sample at ninety degrees to one another. The sample is mounted in a magnetically detachable sample holder which can be positioned horizontally or vertically on the exposure stage, making it easy to carry out sequential orthogonal exposures. The exposure stage is mounted on a rotary stage using four optical posts for stability. An amplified photodiode sits in front of and just below the exposure stage, providing a relative measure of the laser intensity

A simple schematic of the complete set-up we use is shown in Fig. 2, see also Table S1 in the supporting information for a complete bill of materials. In brief, light from a fibre-coupled laser passes through a Half-Wave Plate (HWP), and then expands for a distance of at least 1 m before reaching a rotatable exposure stage that holds a plane-mirror and the sample at $90^\circ$ to each other. Part of the laser beam strikes the sample directly, while another part first strikes the mirror and then reflects onto the sample. The two beams overlap, generating an interference pattern. An optical shutter between the fibre tip and the HWP is used to open and close the laser beam. An amplified photodiode is positioned below and just in front of the exposure stage to monitor the intensity of the laser light. All components are mounted on a stable optical breadboard.

#### Light-source and associated optics

The light-source is a 405-nm, 20-mW, fibre-coupled single-longitudinal mode laser (0405L-25A-NI-AT-NF, Integrated Optics Co.), equipped with a polarisation-maintaining (PM) single-mode fibre (S405-XP). Use of a PM fibre is important to minimise drift in the polarisation state due to temperature changes or mechanical stresses. The laser uses thermo-electric cooling rather than a fan to minimise vibrations and is mounted on a large aluminium plate, using thermal paste to maximise heat-dissipation. The manufacturer quotes a spectral linewidth of <0.018 nm for the laser, measured using an optical spectrum analyser (OSA). This corresponds to a coherence length of around 9 mm at 405 nm—sufficient to maintain an interference pattern that extends over several millimetres. Manufacturers such as Crystalaser and Hübner Photonics supply fibre-coupled lasers with coherence lengths of several metres that allow patterning over much larger areas, albeit at substantially higher cost.

**Fig. 3:**
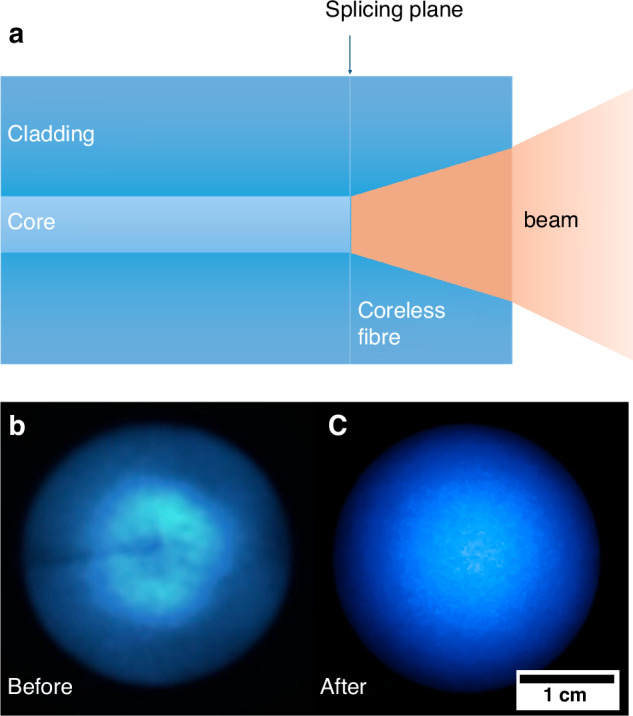
Characteristics of fibre optic. **a** Schematic showing end-cap termination of the SMF. A short length of coreless fibre is spliced onto the end of the SMF, allowing the beam to partially expand before reaching the glass/air interface, reducing the intensity at the tip and hence reducing the tendency for burn-on of debris. **b**, **c** Images of laser spot on a white-screen before and after cleaning the end-cap. Maintaining a clean, debris-free laser tip is far easier when the SMF is terminated with a coreless endcap. Images were recorded ~50 cm away from the fibre tip

The laser fibre is terminated with an end-cap—a short (sub-mm) length of coreless fibre that is fusion-spliced to the end of the SMF, allowing the beam to expand before it reaches the glass/air boundary (Fig. 3a). The end-cap has two main benefits: firstly, the lower intensity of the expanded beam as it leaves the fibre reduces the risk of electrostatically attracted dust in the air from being burnt onto the tip and introducing spatial noise into the beam profile; and, secondly, having an end-cap allows dust, dirt or burnt-on debris to be removed from the tip without damaging the core-cladding interface. Periodic cleaning of the end-cap with a lint-free wipe moistened with isopropanol or a similar solvent is essential to ensure a clean output beam. Fig 3b, c show the beam-profile observed on a sheet of white paper (50 cm from the fibre-tip) before and after cleaning the end-cap. The uncleaned fibre yields a non-uniform beam with a high degree of spatial noise, while the cleaned fibre yields the expected, structureless Gaussian-like profile. The numerical aperture of the fibre is ~0.12, implying a radial divergence of 1.2 mm for every 10 mm travelled by the laser through free-space.

**Fig. 4:**
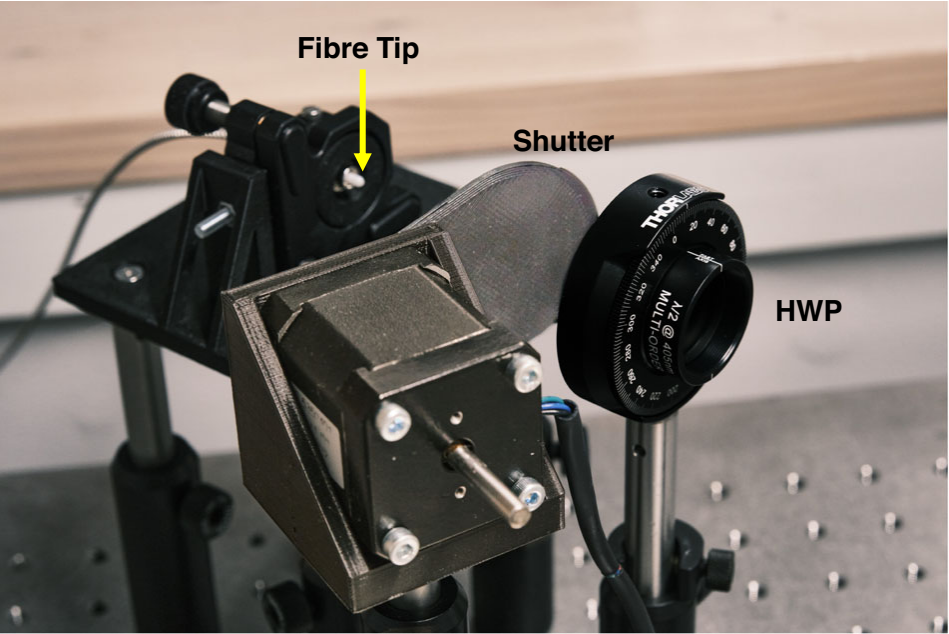
Close-up photograph of fibre tip holder and adjacent optics. Photograph shows fibre tip secured in a 3D-printed mount, motor-driven shutter, and half-waveplate (HWP) in an indexed rotation mount for controlling the polarisation axis of the laser, see also Fig. S2

In our set-up, the tip is held in a simple 3D-printed cylindrical tip-holder (Fig. 4 and Fig. S2a, b) that prevents movement of the tip without creating unwanted stresses that might otherwise affect the polarisation of the output beam. The tip-holder is inserted into a kinematic mount, which allows the direction of the beam axis to be adjusted and aligned to the corner of the exposure stage where the mirror and sample holder meet (see below). The kinematic mount is attached to a 3D-printed baseplate that in turn is fixed to an optical breadboard using four optical posts for maximum stability (Fig. S2c), with the fibre-tip sitting at the same height as the centre of the sample.

Just after the fibre-tip, there is a simple shutter formed from a small 3D-printed, paddle-shaped blade, which rotates out of and into the beam-path to start and stop the exposure (Fig. S3). The paddle is attached to the shaft of a standard NEMA 17 type stepper motor, powered by an inexpensive off-the-shelf stepper driver (DRV8825). The driver responds to step and direction signals from a microcontroller, and is set to a fairly high level of microstepping (32 steps per full step in our set-up) to minimise noise and vibration that would otherwise disrupt the interference pattern at the start and end of the exposure.

Positioned after the shutter is an optional half-wave plate (HWP, Thorlabs WPH10M) in an indexed rotation mount that can be used to adjust the polarisation axis of the divergent beam. To maximise contrast in the interference pattern, the directly incident beam and the reflected beam must have matching polarisation axes. In our set-up, the mirror is oriented vertically and we set the polarisation axis of the (horizontal) laser-beam to vertical too, i.e. we use S-polarised light that remains vertically polarised under reflection by the vertical mirror. (P-polarised light also retains its horizontal polarisation under reflection by a vertical mirror, but S-polarised light is preferred for LM-LIL as metallic mirrors typically show slightly higher reflectivity towards S-polarised light). Turning the HWP through an angle of *θ* rotates the polarisation axis by an angle 2*θ*, allowing it to be brought into the required vertical orientation. Alignment of the HWP may be carried out by (temporarily) placing a linear polariser with a horizontally aligned polarisation axis in the beam-path and rotating the HWP until maximum extinction is achieved. (A simple method for aligning the linear polariser to the horizontal axis may be found in ref. ^34^).

In set-ups that use a free-space laser and fibre coupler, the HWP may be placed before the laser beam enters the fibre, which means it does not introduce any spatial noise into the beam (since noise due to the HWP is absorbed by the fibre-cladding). Our use of a pigtailed laser means we are unfortunately forced to place the HWP after the fibre output where it *can* introduce spatial noise. It is important, therefore, that the HWP is free from scratches and cleaned on a regular basis. For particularly demanding applications, the HWP may be omitted from the set-up altogether, and the fibre-tip itself may be rotated to bring the polarisation axis into the required vertical alignment, taking care not to damage the fibre in the process (If the fibre is damaged, the laser must be sent back to the manufacturer for repair or replacement, so we prefer to use an HWP) (Fig 4).

#### Exposure stage

**Fig. 5:**
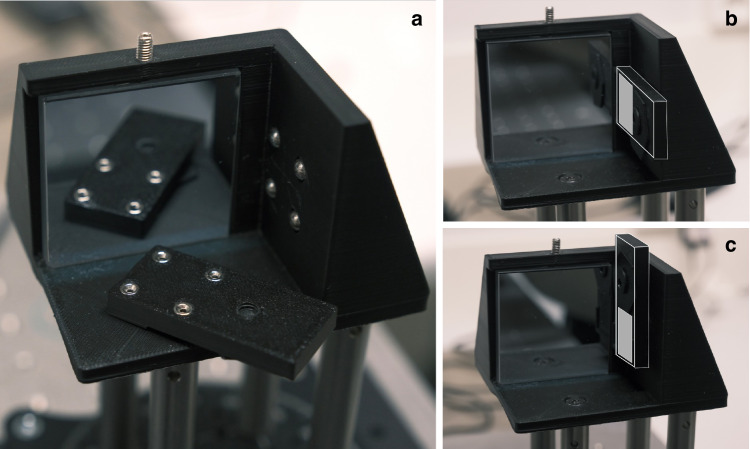
Close-up photographs of the exposure stage. **a** Exposure stage with sample holder detached from the wall; rear-side of the sample holder is exposed with four cylindrical magnets visible; four ferromagnetic steel balls are partially inset into the wall of the exposure stage. **b**, **c** Exposure holder with sample holder attached in its two orthogonal positions; the edges of the sample holder have been highlighted in white for better visibility. See also Fig. S4

The sample and mirror are mounted at 90° to each other on orthogonal walls of a rigid, 3D-printed exposure stage (Fig. 5 and S4). The mirror sits snugly in a square-shaped recess in one wall of the mount, held in place by a vertical grub-screw, while the sample sits in a detachable sample holder that clamps magnetically to the other wall. When in place, the sample sits close to the mirror with a <1-mm gap between the sample edge and the face of the mirror. The exposure stage is mounted on a manually operated precision rotation stage (RP-03/M) using four optical posts (for stability), allowing the sample and mirror to be jointly rotated with respect to the beam axis. The corner where the sample and mirror meet is positioned at the centre of the rotation stage. When the stage is in its home position, the sample and mirror are oriented at ~±45° with respect to the beam axis (treating the wavefront as roughly planar) and the direct and reflected beams strike the sample at equal angles of ~±45° (Fig. 1c). Turning the stage anticlockwise from its home position causes the angle between the direct beam and the reflected beam to decrease (Fig. 1d), and leads to an increase in the pitch of the interference pattern.

When the direct and indirect beams strike the sample at angles $\pm \theta$, the pitch size *p* of the resulting interference pattern is given by

1 $$p=\frac{\lambda }{2\sin \theta }$$

where $\lambda$ is the laser-wavelength^35^.

The (roughly) Gaussian variation in laser-beam intensity in the transverse direction means different parts of the sample receive different exposure doses, so that some parts of the sample are overexposed while other parts are underexposed. This leads in turn to inconsistent development during sample processing, resulting for instance in variable line-widths across the sample. To ensure a more uniform exposure, the exposure stage should be placed a good distance (preferably several metres) from the fibre-tip.

#### Mirror

The choice of mirror is critical. It should: (i) be front-reflecting to prevent ghost reflections; (ii) have a tight optical flatness tolerance (ideally $\lambda /8$ or better) to avoid significantly distorting the fringe pattern; (iii) be highly reflective at the laser wavelength since the interfering beams need near-equal intensity to maximise fringe-contrast; and (iv) be substantially larger than the sample to avoid vignetting of the fringe pattern due to clipping of the reflected beam by an undersized mirror. For our set-up, we specifically use a 50-mm × 50-mm UV-enhanced aluminium mirror (PFSQ20-03-F01, Thorlabs Inc.) with $\lambda /8$ optical flatness at 633 nm and a reflectivity of >90% at 405 nm.

#### Sample holder

**Fig. 6:**
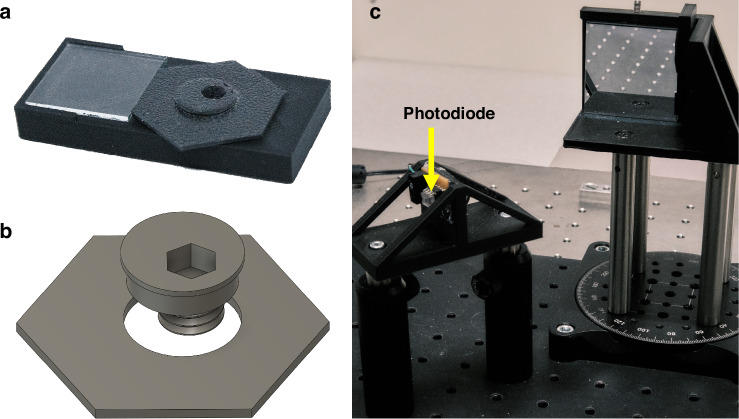
Sample holder and mount for photodiode. **a** Photograph of the top-side of the sample holder, showing a 2-cm by 2-cm glass slide held in place by an eccentric clamp; **b** Schematic of the eccentric clamp formed from a hexagonal nut with a round-hole at its centre and an eccentric bolt in which the head is offset from the shaft; rotating the bolthead clockwise inside the nut causes the nut to push against the side of the sample, holding it in place. **c** Photograph showing the amplified photodiode below and just in front of the exposure stage. Yellow arrow indicates the location of the photodiode

The sample holder is a simple 3D-printed rectangular piece, equipped with an eccentric-nut-style clamp to gently hold the sample in place by its edge (Fig. 6b). The part is designed to hold a 20-mm by 20-mm sample of thickness <1-mm, although it could be straightforwardly adapted for other sample sizes. We consider a 20-mm by 20-mm sample size to be a sensible upper limit with our current set-up due to the Gaussian intensity profile of the light from the fibre. With substantially larger substrates, excessive variations in exposure intensity across the sample would lead to non-uniform patterning.

Set into the underside of the sample holder are four cylindrical neodymium magnets, arranged in a square formation (see Fig. 5a). The magnets have a conical recess at one end and mate with four ferromagnetic steel balls that are partially inset into one wall of the exposure stage. When the sample holder is brought into approximately the correct position, it snaps into place under the magnetic pull of the cylindrical magnets. The magnets automatically centre themselves on the steel balls during this process, ensuring good repeatability in positioning. The square layout of the steel balls and the cylindrical magnets means the sample holder can attach in orthogonal directions, allowing for the straightforward fabrication of 2D arrays by sequential exposures at 90° relative sample orientations. In both orientations the sample holder is held firmly against the exposure stage wall by the strong (N52) cylindrical magnets, with no relative movement between the two to disrupt the fringe pattern.

#### Exposure monitoring

An amplified photodiode is placed in the beam-path close to the exposure stage (Fig. 6c) to obtain a relative measure of the intensity of light falling on the sample during the exposure. The photodiode is mounted on a rigid 3D-printed mount that is secured to the optical breadboard by two posts for stability (Fig. S5). The signal from the photodiode is read by a 24-bit analogue-to-digit converter board (ADC 6 Click, MikroElektronika d.o.o.), which is connected to a microcontroller (the same one that is used to control the stepper motor). A software programme controls the duration of the exposure to ensure that the integrated exposure dose is consistent from one run to the next (see section entitled ‘Exposure Procedure’).

#### Vibration isolation

Although the Lloyd’s mirror configuration is less sensitive to vibration than multiple-beam interference lithography, efforts should nevertheless be taken to minimise vibration. Ideally, the set-up should be installed in the basement of a building far away from potential disturbances (trainlines, road-traffic etc). Our own set-up, however, is located on the second floor of a large building. Everything is mounted on a standard 1.5-m by 1-m by 7.5-cm honeycomb breadboard that sits on a non-isolating support-frame. 1-cm-thick foam pads are inserted under the foot of each leg to provide modest vibration damping. The breadboard was one that happened to be available to use at the time the system was set-up, and is no sense an optimal choice. A larger, sturdier table with better vibration isolation would certainly be preferable. However, with the current breadboard—even in its sub-optimal second-floor location—we are nonetheless able to achieve high-quality arrays.

#### Air-flow management

Air-flow is often a greater concern than vibration when it comes to LM-LIL since the rigid attachment of the mirror and sample does little to mitigate the disruptive effects of air-flow on the interference pattern. (Air-currents cause localised changes in temperature and pressure that affect the refractive index and hence alter the optical path difference between the interfering beams, leading to drift in the fringe-pattern). Placing the set-up in a moderately air-tight enclosure is therefore important. Our set-up is covered by a large 1.5-m by 1-m by 0.6-m box (Fig. S6). The frame of the box is made from aluminium extrusion, and the roof, side-walls and rear-wall of the box are formed from opaque 4-mm-thick panels of polyvinyl chloride (PVC), which lend rigidity to the enclosure. The interior is spray-coated with matt-black paint to reduce stray reflections. The base of the frame is secured to the optical breadboard using screws and simple 3D-printed brackets. For convenience, we use overlapping PVC strip-curtains for the front of the box, as they provide easy physical access to the set-up when needed, but form a decent (albeit imperfect) seal against outside air when hanging. Owing to their relatively short length, the curtains have a slight tendency to curl-up, creating unwanted air-gaps. Magnetic clasps at the bottom of each curtain are therefore used to hold the curtains securely against the frame of the box. The transparent strip-curtains are convenient as they allow the user to have a clear view of the set-up at all times, although they could potentially cause problematic stray reflections; if necessary, they could be replaced by matt-black fabric curtains.

It is important to stress that, although using an enclosure provides considerable protection against air-flow, it is not a perfect solution. Therefore, the set-up should not be placed in a room with strong draughts or aggressive air-conditioning, and any electrical items with fan-cooling that could disrupt the air-flow should be switched off before carrying out any exposures. It is also important to keep movement in the room to a minimum and to allow sufficient time after loading the sample and closing the curtains for the air to stabilise before beginning an exposure. (Best practice is to leave the room some minutes before remotely activating the exposure). Baffles may be inserted inside the box to reduce interior air-flow, although we do not use them in our current set-up.

#### Interferometric stability testing

The degree to which vibration and air-flow must be mitigated depends on the environment where the set-up will operate, and it is therefore important to have some means of testing whether the chosen mitigation strategies are sufficient—preferably a simple test that can be run every time an exposure is carried out. As the Lloyd’s mirror fringe-pattern is too fine to observe directly, we have built into our set-up a Michelson interferometer^36^ that is permanently mounted on the optical breadboard inside the enclosure. In contrast to the Lloyd’s mirror configuration, the interfering beams in a Michelson interferometer are co-axial, which results in large fringe-spacing that can easily be viewed by eye. The Michelson architecture is more sensitive to the disruptive effects of vibration and air-flow than the Lloyd’s mirror configuration because—as with MB-LIL—the two beams follow spatially separated optical paths. The successful observation of a static fringe pattern on a Michelson interferometer can therefore be taken as strong evidence that the environment is stable enough for successful LM-LIL. The time taken to recover a stable interference pattern after a disturbance provides useful information about the recovery time of the set-up. Ideally, a stable fringe-pattern should re-establish itself on the interferometer within a second or two of the disturbance ending. If this is not the case, the system is underdamped with respect to vibration and should be adjusted accordingly.

**Fig. 7:**
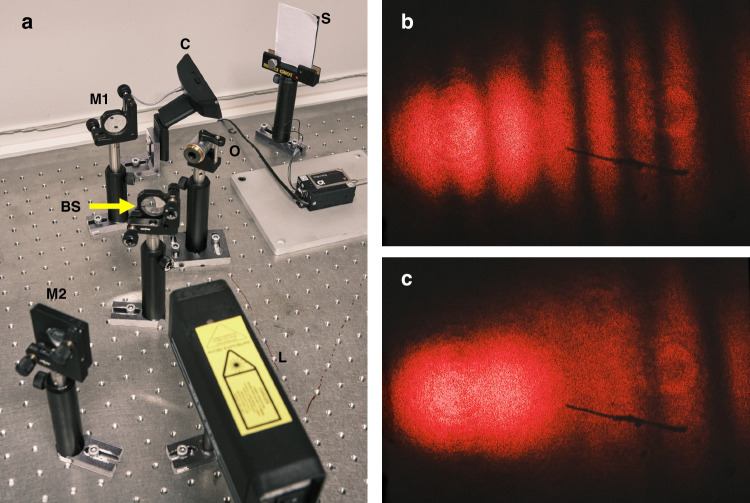
Michelson interferometer and interference patterns obtained under stationary and vibrating conditions. **a** In-situ Michelson interferometer, comprising HeNe laser (L), beam-splitter (BS), two mirrors (M1, M2), microscope objective (O), white screen (S) and digital camera (C). **b**, **c** Interference pattern observed on screen under stable conditions (**b**) and following a disturbance (**c**). See also Fig. S7

We use a standard Michelson configuration (Fig. 7a, S7) in our set-up with a small 633-nm HeNe laser, a 50:50 beam-splitter, a mirror in each beam-path, and a reversed microscope objective to project an expanded image of the fringe pattern onto a white paper screen. The only deviation from the text-book Michelson configuration is our use of non-orthogonal beam-paths^36^, which makes it somewhat easier to pass the beams through the beam-splitter without clipping. A simple webcam-style digital camera is used to monitor the interference pattern on the paper screen (Fig. 7b). Air flow due to draughts leads to continuous drift in the interferometer fringe pattern, and must be stringently avoided using the precautions described above. When these precautions are followed, the interferometer fringe pattern typically stabilises within 30 min of switching on the HeNe laser, and remains broadly static thereafter with only minor wander due to residual air currents, temperature fluctuations or frequency drift in the HeNe laser, see Video S1. Experience shows that, with the observed level of stability in the interferometer fringe pattern, high-quality sub-250 nm patterning of a photoresist is routinely achievable using the Lloyd’s mirror lithography system. Immediately after a mechanical disturbance, the interferometer fringe pattern is visibly blurred (Fig. 7c), but a stable fringe pattern is restored within ~2 s. (Video S2 shows the effect on the interferometer fringe pattern of repeatedly knocking the optical table).

#### Environmental control

Our set-up is located in a small room that lacks built-in temperature-control, humidity-control, or air-filtration. We currently take no specific precautions to control temperature or humidity since indoor temperatures and humidity levels are typically low where the set-up is located (Norway). However, the room is periodically cleaned to remove accumulated dust, and we use a self-standing domestic air-purifier (Mill Silent Pro compact) equipped with a HEPA-13 medical grade filter to extract dust from the air. The air-purifier is left running 24 h a day to minimise dust build-up. The unit features a boost mode, which we activate for a short period before the set-up is due to be used to further reduce dust levels in the air. The air-purifier is always paused before any exposures are carried out to minimise air-flow. The room has a large window, which is blacked-out using aluminium foil. While we obtain satisfactory results carrying out exposures in this room, for demanding applications, it would be preferable to locate the set-up in a Class ISO-5 cleanroom.

### Sample handling

All sample processing before and after exposure is carried out in a Class ISO-6 (1000) and ISO-5 (100) cleanroom, while sample exposure is carried out in a separate room using the LIL set-up described above.

## Pre-exposure sample preparation

### Cleaning

Samples are prepared on 2-cm by 2-cm glass or silicon substrates. Prior to use, each substrate is rinsed thoroughly in acetone and isopropanol and then washed in distilled water, before drying with a nitrogen spray gun. If the substrate has been used previously, it is also subjected to an O_2_-plasma treatment for 3 min (100 W, O_2_ flow rate: 5 mL min^−1^). The substrate is then placed on a 95 °C hotplate and baked for 1 min in air to drive off any left-over solvent. It is then removed from the hotplate and allowed to cool to room temperature.

### Application of antireflection coating

An antireflection coating is used to suppress reflections from the substrate that would otherwise create unwanted standing waves, distorting both the exposure pattern and the final edge-profile of the patterned features. 200 nm of a bottom antireflective layer coating (BARC, AZ Barli II 200, MicroChemicals GmbH) is deposited on the substrate by spin-coating at 4000 rpm for 60 s. The substrate is then transferred to a 200 °C hotplate for 1 min. It is then removed from the hotplate and allowed to cool to room temperature. To avoid coating defects in the resist layer when it is deposited on top of the BARC, the manufacturer recommends that the BARC should be deposited at a high spin-speed (>3500 rpm).

### Application of photoresist

The photoresist layer is deposited on top of the BARC. A wide variety of positive and negative photoresists may be used, but we have found that the positive photoresists ma-P 1275 and mr-P 1200LIL from Micro Resist Technology GmbH provide good results:

- **ma-P 1275** is a general-purpose photoresist that must be diluted in ma-T 1050 thinner at a 1:3 ratio (photoresist: thinner) before use to achieve films that are sufficiently thin for LIL. A 300-nm layer of Ma-P 1275 is obtained by spin-coating the diluted ma-P 1275 at 6000 rpm for 40 s, and then soft-baking at 105 °C for 1 min.
- **mr-P 1200LIL** is a specialist, fine-resolution photoresist specifically developed for laser interference lithography, capable of producing features with steep vertical walls. A 200-nm layer is obtained by spin-coating the as-received photoresist at 3000 rpm for 30 s, and then soft-baking at 105 °C for 1 min.

In our experience both photoresists are capable of yielding high quality arrays via LM-LIL, although mr-P 1200LIL has the advantage that it may be used directly without dilution, eliminating a possible source of experimental variability.

After deposition of the photoresist, the samples are stored in an air-tight, foil-covered sample-case. Exposures are carried out within 1 h of photoresist deposition.

### Exposure procedure

Each time the LIL system is used, the strip curtains of the enclosure are opened, the optics are checked for cleanliness and cleaned if necessary using lens wipes. At least 1 h before we are due to carry out an exposure, the fibre-coupled laser and the HeNe laser on the interferometer are both switched on to allow sufficient time for them to stabilise.

With the shutter open, the stability of the beam from the fibre laser is checked by monitoring the signal from the photodiode. The signal strength is checked against historic values to verify the set-up is performing as expected. A lower signal than expected suggests misalignment of the system, unclean optics, or a problem with the laser. The uniformity of the diverging beam from the fibre laser is checked using a sheet of white paper. The beam should show no spatial structure; if it does, the laser is switched off and further cleaning is carried out, paying particular attention to the fibre tip. The shutter is then returned to the closed position.

With the enclosure still open, the air-filtration unit is switched from its normal operating mode to boost mode to further reduce dust levels in the air. After 30 min or so in boost mode, the unit is turned off to minimise air flow. The room-lights are switched off, and a dim, orange sidelight is switched on to aid visibility. A sample is loaded into the sample-holder, and the holder is attached magnetically to the exposure stage. The strip curtains are closed, and at least 1 min is allowed to elapse before proceeding further to allow the air to stabilise. The image on the paper screen of the interferometer is checked to ensure it is showing clear, stable fringes.

A simple computer programme that displays a live-stream of the interferometer fringe pattern and communicates with the microcontroller and the laser is used to carry out the exposure. The programme may be activated remotely by a web-based interface, so the user can leave the room to minimise vibrations and air-flow during exposure. There are two selectable operating modes—‘time-mode’ where the sample is exposed for a specified period of time, and ‘dose-mode’ where the exposure continues until the cumulated dose reaches a specified value. Dose mode is the mode we typically use as it automatically compensates for changes in laser intensity, ensuring the same exposure dose every time. Regardless of the mode selected, when the programme is run, the photodiode signal is recorded for 10 s before the shutter is opened, allowing a reliable measurement of the background signal to be obtained. When the shutter opens, the cumulated dose is calculated by subtracting the background signal from the measured signal and integrating the corrected signal with respect to time. When the target time or dose is reached (depending on the selected mode), the shutter closes and the photodiode signal is recorded for a further 10 s before the programme ends. Hence, a background signal is obtained both before and after the exposure. If the system is stable, the two sets of background signals should be consistent. A text file of the photodiode signal versus time and a video-recording of the interferometer fringe-pattern over the course of the exposure is saved automatically for diagnostic purposes. The process can then be repeated at a different sample orientation or with different samples, adjusting the target time/dose as necessary. The required time/dose must be determined empirically for the specific hardware, layout, materials and photolithography processes being employed.

When the series of exposures have been completed, the lasers are switched off, the strip-curtains are closed, and the air-filtration unit is returned to its normal operating mode. (The door to the room is kept closed at all times to ensure effective dust removal). Samples are returned to the sample case for transportation back to the cleanroom for further processing.

## Post-exposure sample-processing

No post-exposure baking is needed for either photoresist.

- **ma-P 1275** is developed for 120 s in a diluted NaOH-based developer ma-D 332/S. The developer is diluted with deionised water (2:1 developer-to-water ratio) to increase the development contrast, i.e. to increase the dissolution rate of exposed resist relative to that of unexposed resist. The sample sits on a wafer dipper and is moved around slowly in the developer to prevent local depletion of active developer; the sample is not shaken as this can lead to collapse of the grating walls. As the photoresist dissolves, a thin streak of coloured liquid (which appears dark-red under the yellow cleanroom lighting) can be seen leaving the sample, reminiscent of wisps of smoke. The immersed sample becomes increasingly iridescent as the development proceeds. Loss of iridescence indicates over-exposure of the sample or over-development. Once the target development time is reached, the sample is removed from the developer and rinsed gently but thoroughly with DI water. The sample is then gently dried using dry nitrogen gas.
- **mr-P 1200LIL** is developed by immersing in ma-D 374/S developer (Micro Resist Technology GmbH) for 20 s. The dissolution of photoresist is less visible for mr-P 1200LIL than for ma-P 1275. In all other respects, the developing process is the same as for ma-P 1275.

Following development, the BARC and the photoresist layers can be anisotropically etched by CHF_3_/O_2_ reactive-ion etching (40 sccm CHF_3_, 10 sccm O_2_, 100 W RF). An etching time of 40 s is sufficient to etch through the exposed BARC and reach the substrate.

### Illustrative results

#### 1D arrays on glass

**Fig. 8:**
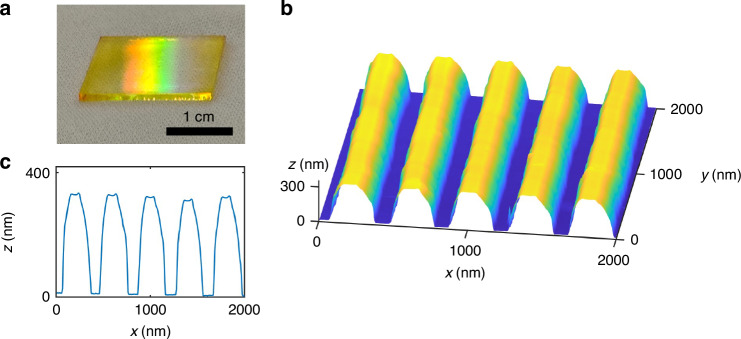
Photograph and AFM imaging of a typical 1D grating. **a** Photograph of a typical grating structure fabricated using a 200-nm base-layer of AZ Barli II 200 BARC followed by a 300-nm layer of the general-purpose positive photoresist Ma-P 1275 after reactive ion etching. **b** 2D AFM profile image of the grating, obtained in tapping mode. **c** AFM line-profile, extracted from (**b**) at *y* = 1000 nm

Figure 8a shows a photograph of a typical 1D-grating on a 2-cm by 2-cm glass substrate, taken under normal room lighting. The sample was fabricated with a 200-nm base-layer of AZ Barli II 200 BARC followed by a 300-nm layer of the general-purpose positive photoresist ma-P 1275, using a single exposure of duration 20 s at incident angles of $\theta =\pm 30^\circ$. The sample was subsequently subjected to reactive ion etching to expose the substrate. The orange colouration of the sample is due to the underlying layer of BARC, while the spectral dispersion is due to diffraction of the white overhead lighting by the grating structure. Figure 8b shows a 2-μm by 2-μm AFM profile of the grating around the centre of the sample, with the flat base of the substrate clearly evident. The 350-nm height of the grating corresponds to the 200-nm height of the BARC layer plus ~150-nm of the overlying photoresist layer, which has been partially etched away. The pitch of the sample is ~400 nm. Figure 8c shows a line profile through the centre of the substrate (*y* = 1000 nm), perpendicular to the grating direction. The steepness of the walls in the lower part of the grating is attributable to the anisotropic nature of the RIE process, which etches the BARC in a near-vertical manner.

**Fig. 9:**
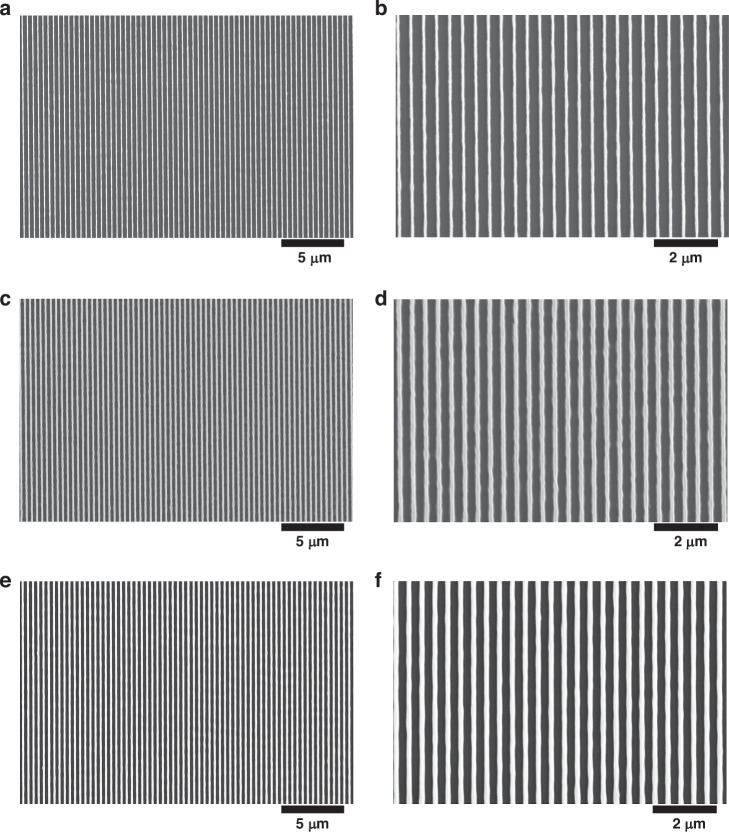
SEM images of a typical 1D grating before and after reactive ion etching. **a**–**d** SEM images of unetched BARC/ma-P 1275 sample obtained close to the centre of the beam (**a**, **b**) and close to the sample centre (**c**, **d**). **e**, **f** SEM images of an equivalent sample after reactive ion etching, measured close to the sample centre. Samples were sputter coated with a 5-nm layer of chromium for imaging

Figure 9a, b show for two different levels of magnification SEM images of a sample (equivalent to the one in Fig. 8) that has been developed but not etched. The images were recorded close to the centre of the beam where the fringe contrast is at its highest, using a 5-nm conducting overlayer of chromium. The grating structure shows high uniformity with a pitch of 406 nm and a linewidth of around 80 nm. Figure 9c, d shows images of the same sample obtained close to the sample centre, where the grating has a similar pitch of around 406 nm but a substantially wider line-width of 130 nm due to the reduced beam intensity. Increasing the clearance between the sample tip and the exposure stage would reduce this variation, leading to a more uniform grating structure across the sample (although a longer exposure time would be needed to compensate for the reduced beam intensity at the sample). Figure 9e, f shows images of an etched sample obtained close to the centre of the sample. The line-width is broadly the same as for the unetched sample in Fig. 9c, d, but the image contrast is enhanced due to the increased grating amplitude after etching.

#### 1D arrays on silicon

**Fig. 10:**
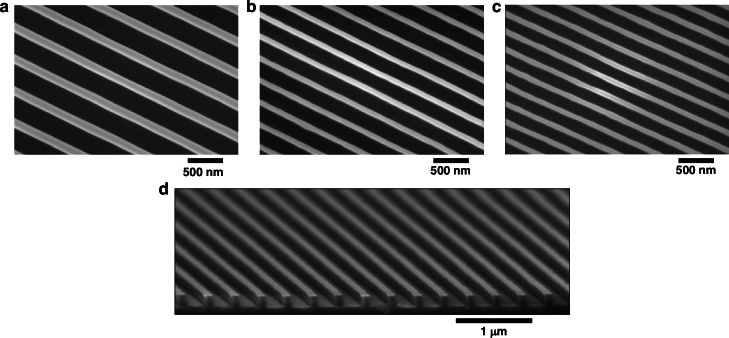
SEM images showing the effect of rotation stage orientation on 1D grating pitch. **a**–**c** SEM images of unetched BARC/mr-P 1200LIL samples, obtained at rotation-stage angles of −15° (**a**), 0° (**b**) and +15° (**c**), corresponding to approximate incidence angles of ±60°, ±45° and ±30°, respectively. **d** Tilted cross-sectional SEM image of an equivalent sample, exposed at an incident angle of ±30° and measured close to the sample centre. Samples were sputter coated with a 5-nm layer of chromium for imaging

Figure 10 shows a series of SEM images for an unetched sample on silicon with a 200-nm base-layer of AZ Barli II 200 BARC, followed by a 200-nm layer of the specialist LIL photoresist mr-P 1200LIL. The samples were prepared using an approximate exposure time of 60 s at rotation-stage angles of −15°, 0° (home) and +15°, corresponding to approximate incidence angles of ±60°, ±45° and ±30°. The corresponding pitch sizes are 218 nm, 273 nm and 395 nm, compared to expected values of 234 nm, 286 nm and 405 nm for ideal planewave illumination. The slight mismatch is attributable to the spherical nature of the wavefront. Increasing the clearance between the sample tip and the exposure stage would result in closer agreement. Figure 10d shows a tilted cross-sectional SEM image of an equivalent sample, exposed at an incident angle of ±30° providing a pitch of ~395 nm. To make the cross-section, a light scratch was made along the centre of the sample with the aid of a diamond scribe, and gentle thumb-pressure was applied to break the wafer cleanly along the scribe line. The image shows good line-uniformity and wall-quality over the image area with respect to both height and edge-profile.

#### 2D arrays on glass

**Fig. 11:**
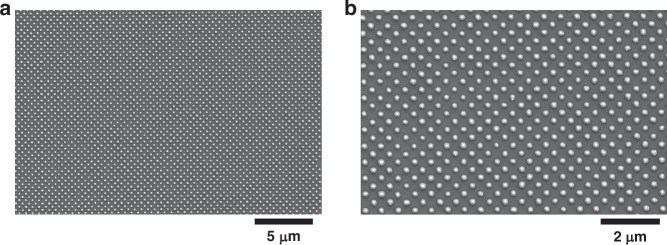
SEM images of an etched 2D array at two levels of magnification. **a** Wide-field; **b** zoomed. The arrays were formed by carrying out two exposures of BARC/ma-P 1275 at 90° relative orientation. The sample was sputter-coated with a 5-nm overlayer of chromium for imaging

Figure 11 shows SEM images of a 2D array fabricated by carrying out two exposures of a BARC/ma-P 1275 sample on glass at 90° relative orientations. (This is straightforward due to the design of the sample holder, which locks into place in both the horizontal and vertical positions). An exposure time of ~13 s was used for each exposure, slightly shorter than the 20-s exposure time used for the 1D arrays with ma-P 1275. The angle of the rotation stage was set to 15°, corresponding to incident angles of ~±30° on the sample. The double exposure generates vertical and horizontal strips of exposed photoresist, which dissolve away during the development stage, leaving approximately circular regions of unexposed photoresist behind on the substrate. The double exposure yields a regular array of spots at the local level, although some further optimisation of the exposure conditions is needed to achieve a more uniform spot shape.

## Conclusion

In conclusion, we have described a simple but effective set-up for Lloyd’s mirror interference lithography, using a 405-nm, fibre-coupled single-frequency laser, a plane mirror and an optional HWP. Use of a pigtailed SMF laser greatly simplifies experimental implementation by eliminating the need to manually align a free-space laser beam to a spatial filter or external fibre, while providing high stability in beam characteristics. Since the laser and fibre are permanently bonded after factory alignment, coupling efficiencies into the fibre remain high even in the presence of environmental perturbations. Free-space coupling systems, by contrast, are highly susceptible to mechanical vibrations, thermal drift and beam pointing variations that degrade the precise alignment required for efficient single-mode fibre coupling. Hence, the proposed set-up retains the advantages reported for previous SMF-based set-ups^27,28^, while addressing their known susceptibility to environmental drift. The termination of the SMF with a coreless end-cap is a small, but important, detail that minimises burnt-on debris, allowing easy maintenance of the tip and ensuring clean, aberration-free illumination.

The system reported here was designed to a tight budget of ~10,000 Euros, most of which went towards the fibre-coupled laser (€6500), HWP (€700), rotational stage (€300) and various mounts and clamps, see bill of materials in Table S1. While the set-up should ideally be located inside a cleanroom with proper vibration isolation and environmental control, it can nonetheless give acceptable results in a bare room, providing care is taken to keep vibrations, air-flow and dust levels to a minimum. Although we use an air-purifier to extract dust, we currently use no other form of environmental control. It would be sensible to install a dehumidifier and a small air-conditioning unit in the room where the set-up is installed (temporarily deactivating them before carrying out exposures).

The biggest limitation of our current set-up is a purely geometric one. Due to space constraints, the clearance between the fibre-tip and the sample is approximately one metre, but a clearance of several metres would be preferable to ensure a more uniform illumination of the sample, and to reduce hyperbolic distortion of the interference pattern. The main price to be paid for this change would be an increase in the exposure time to compensate for the reduced light-intensity at the sample. Alternatively, it would be possible to use beam-shaping optics^37^ to collimate the spherical wavefront and modify the intensity distribution from Gaussian to top-hat (flat). This would allow the current footprint to be used more effectively, albeit with the inevitable introduction of some extra spatial noise into the fringe pattern (and a likely doubling of the build cost for high-quality optics). Hence, there is a trade-off between simplicity of design and affordability (as favoured in the current implementation) versus uniformity and linearity in the fringe profile (which would be maximised using collimated top-hat illumination).

Even in its current form, however, we have found that the set-up reported here provides a practical, versatile and cost-effective system for nanoscale patterning over macroscopic length-scales that is sufficient for many applications in nanoscale science and technology. It provides excellent resilience to mechanical vibrations, allowing high-quality one- and two-dimensional arrays to be obtained in mechanically noisy environments where multiple-beam approaches would fail without dynamic fringe-locking. We consider the streamlined experimental arrangement described here—made possible by the use of a fibre-coupled, single-frequency laser—to be an attractive option for anyone wishing to set-up a simple but effective system for Lloyd’s-mirror-based laser interference lithography.

## Supplementary information

Supporting information

Table S1

Video S1

Video S2

## Data Availability

Data files are included as Supporting Information.
