# Supplementary material for: A streamlined set-up for Lloyd’s mirror interference lithography, using a single-mode-fibre-coupled laser
Source: Microsyst Nanoeng. 2026 Jun 10;12:230. doi: 10.1038/s41378-026-01186-4 (PMC13254252; doi:10.1038/s41378-026-01186-4)
Supplement: Supplementary file 1 — Supporting information [file 41378_2026_1186_MOESM1_ESM.pdf]

A streamlined set-up for Lloyd's mirror interference lithography, using a single-mode-fibre-coupled laser

E. Lian, E. Perivolari, Y. Liu and J. C. deMello
john.demello@ntnu.no

Supplementary Information

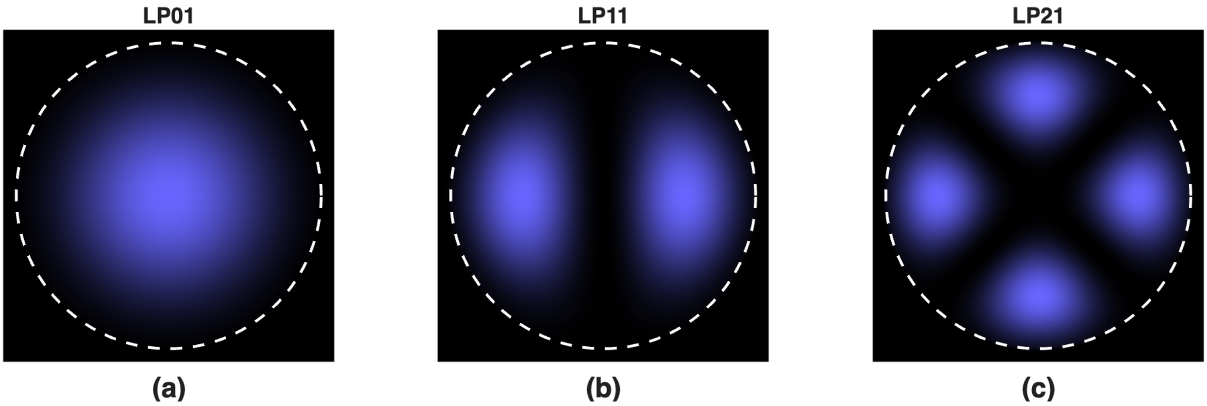

Fig. S1 – Intensity plots for the three lowest-order guided modes in a weakly guiding fibre, assuming a core radius of 12 μm and a wavelength of 405 nm. The white dotted line denotes the boundary between the core and the cladding. The LP01 fundamental mode has a featureless Gaussian-like transverse intensity profile that is well-suited to LM-LIL, whereas higher-order modes are more structured with multiple lobes. In a single mode fibre, only the LP01 mode can propagate. Intensity plots were made using the Fiber modes package by Lucian Bojor (2025). (https://www.mathworks.com/matlabcentral/fileexchange/9126-fiber-modes).

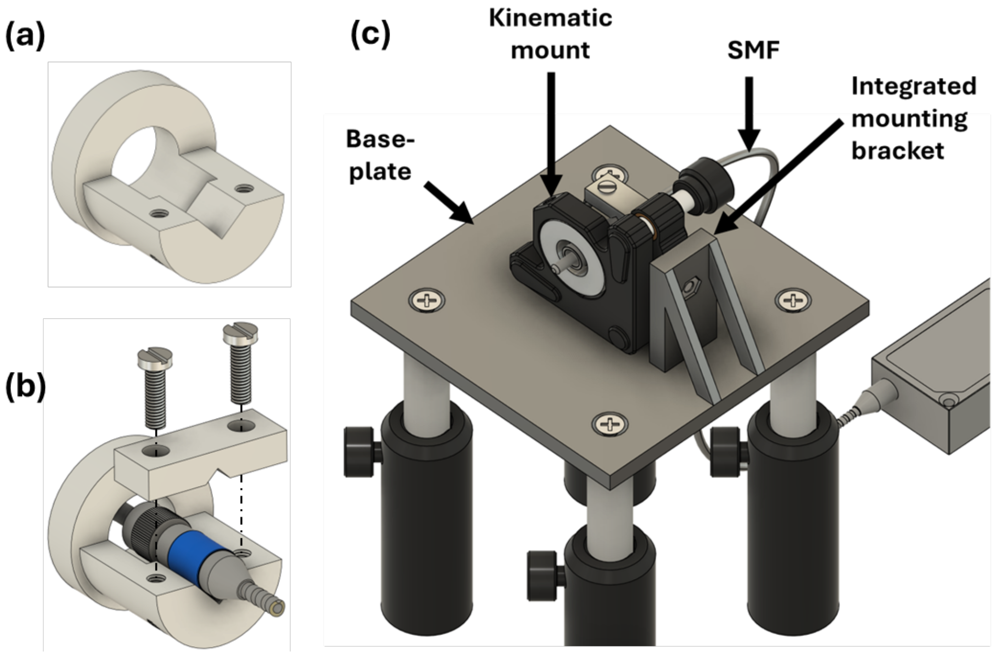

Fig. S2 – Schematic of the 3D-printed support used to hold the fibre-tip without **(a)** and with **(b)** the tip inserted. A rubber sleeve (blue) around the fibre-tip sits in a V-groove in the lower half of the support, and is held in place by a rectangular clamp; the circular front-end of the support fits inside a kinematic mount for 25-mm optics. The kinematic mount is securely attached to a 3D-printed base-plate (**c**) by means of two screw-holes – one in the centre of the base-plate (not visible) and another in an integrated bracket. Four optical posts are used to firmly attach the baseplate to the optical breadboard. The height of the tip matches the centre of the sample. By adjusting the tilt of the kinematic mount, the beam-axis can be aligned to the height of the sample centre and the corner wall of the exposure stage.

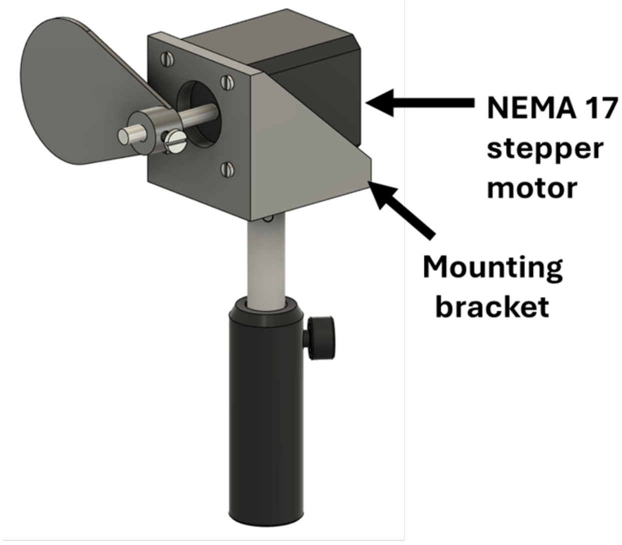

(a)

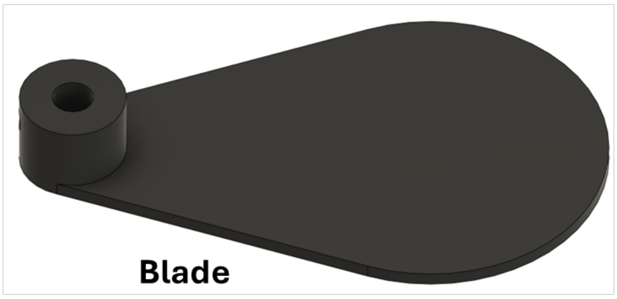

(b)

Fig. S3 – (a) Complete shutter assembly, comprising a 3D-printed beam-stop attached to the rotor of a NEMA 17 stepper motor, a 3D-printed mounting bracket, and an optical post. **(b)** Close-up of the paddle-shaped blade used as a beam-stop. The blade is secured to the shaft of the rotor using a grub-screw.

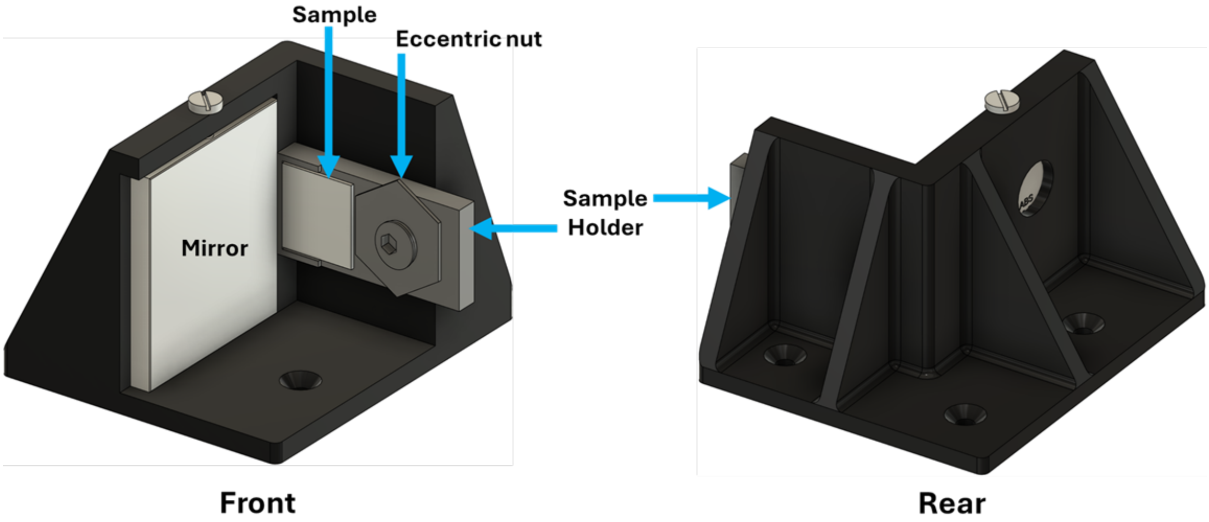

Fig. S4 – Front and rear-views of the 3D-printed exposure stage that is used to mount the mirror and the sample-holder. The mirror is secured with a grub-screw, while the (detachable) sample holder is held in place magnetically. The exposure stage is attached to a rotation stage on the optical breadboard using four optical posts. The centre of the sample is at the same height as the fibre tip. Additional images of the exposure stage are provided in the main paper.

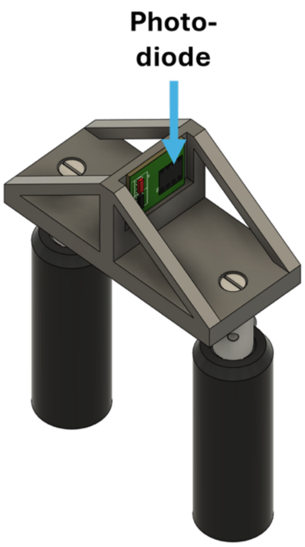

Fig. S5 – Schematic of 3D-printed mount for the amplified photodiode. The photodiode is mounted on a small PCB, which is secured to a vertical wall in the centre of the mount by hot glue. The triangular side-arms increase the rigidity of the mount, and ensure that the PCB is held securely with minimal vibration. The mount sits below the exposure stage in the path of the laser beam, with the photodiode facing towards the fibre-tip. The voltage from the photodiode is read by a 24-bit ADC, and provides a relative measure of the intensity of light falling on the sample.

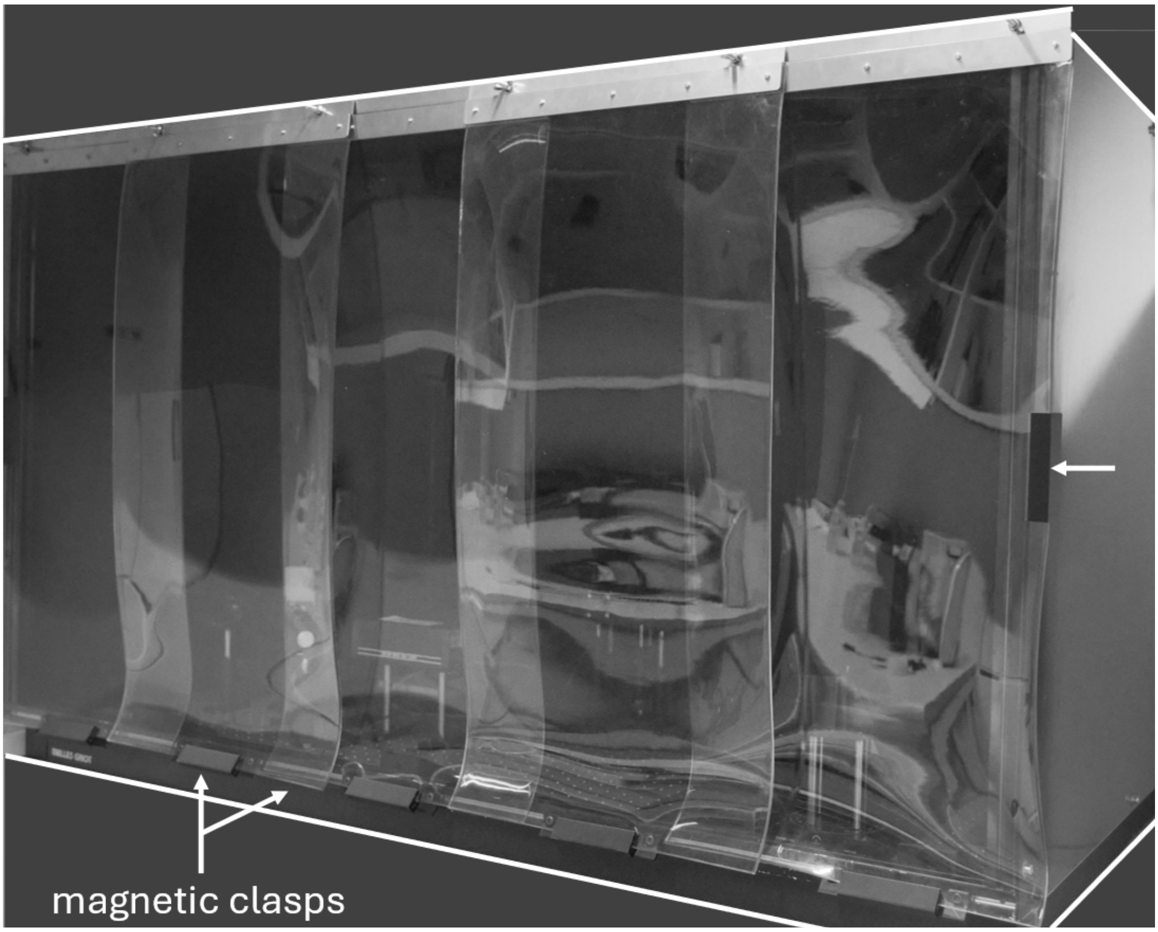

Fig. S6 – Photograph of enclosure used to cover the set-up and minimise air-flow. The frame of the enclosure is made from aluminium extrusion, and it is sealed on the top, back and sides by 4-mm-thick PVC panelling. The frame is screwed to the optical breadboard using 3D-printed brackets. The front of the enclosure is covered with PVC strip-curtains, which provide a fairly good seal against draughts. Magnetic clasps on the bottom and sides hold the curtains in place (white arrows).

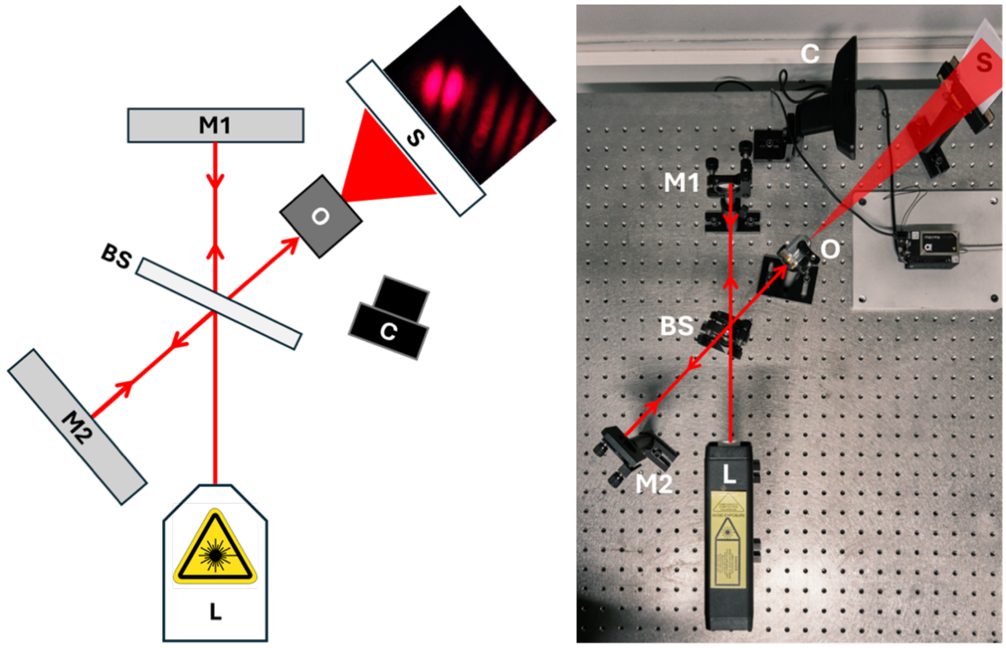

(a) (b)

Fig. S7 – Schematic **(a)** and photograph **(b)** of the in-situ Michelson interferometer. Light from a laser (L) passes through a beam-splitter (BS), and is divided into two beams that are retroreflected by the two mirrors (M1 and M2). The reflected beams return to the beam-splitter and are partially reflected towards the inverted microscope objective (O), which throws a magnified image of the resulting interference pattern onto a white screen (S). The live interference pattern is monitored by a digital camera (C). A non-orthogonal geometry is used for the two light-paths to avoid clipping the walls of the kinematic mount that holds the beam-splitter. Care is taken to avoid retroreflecting laser light back into the laser cavity as this can lead to laser instability. After initial alignment, M1, M2, and the beam splitter are therefore marginally adjusted so that the outgoing beams are no longer exactly collinear, but instead cross and interfere at the objective.
