# Supplementary material for: A streamlined set-up for Lloyd’s mirror interference lithography, using a single-mode-fibre-coupled laser
Source: Microsyst Nanoeng. 2026 Jun 10;12:230. doi: 10.1038/s41378-026-01186-4 (PMC13254252; doi:10.1038/s41378-026-01186-4)
Supplement: Supplementary file 2 — Table S1 [file 41378_2026_1186_MOESM2_ESM.pdf]

Part	Part Name	Supplier	Part ID	Price	Number	Total price (USD)
Optics (Sample Stage & Fibre Stage)	Ø1" Zero-Order Half-Wave Plate	Thorlab	WPH10M-405	666.05	1	666.05
	Ø4.3" Manual Rotation Stage	Thorlab	RP03	335.83	1	335.83
	Ø12.7 mm Optical Post,L=100mm	Thorlab	TR100/M	7.14	10	71.4
	Ø12.7 mm Post Holder	Thorlab	PH75/M	10.29	12	123.48
	2" x 2" Square UV-Enhanced Aluminum Mirrors	Thorlab	PFSQ20-03-F01	144.9	1	144.9
	Ø12.7 mm Optical Post,L=50mm	Thorlab	TR50/M	6.31	2	12.62
	Rotation Mount for Ø1" (Ø25.4 mm) Optics	Thorlab	RSP1X15/M	166.51	1	166.51
	405 nm SLM Laser	Integrated Optics	0405L-25A-NI-AT-NF	6600‡	1	#VALUE!
	Kinematic Mirror Mount for Ø1" Optics	Thorlab	KM100	44.78	1	44.78
Contol Electronics	Wireless Single Board Computer	Raspberry Pi	Raspberry Pi 3 Model B	50	1	50
	MCU (motor control and signal processing)	PJRC	Teensy 4.0	23.8	1	23.8
	Stepper Motor Driver	Polulu Robotics & Electronics	A4988, DRV8825 Stepper Driver	7.2	1	7.2
	External Analog to Digital Convertor	Mikroe	ADC 6 Click	42	1	42
	Photodetector	Texas Instruments	OPT101-p Amplified Si Photodiode	9.06	1	9.06
	Stepper Motor	Zeptobit	NEMA17 stepper motor 40 mm	15	1	15
	DC Power Supply (For Stepper Motor) 220V AC ~ 9V/2A	RockPower	NT 13	12.8	1	12.8
	DC Power Supply (For Main Controller), 220V AC ~ 5V/2A	Delta Power	5V/2A/USB/C Delta Power	5.1	1	5.1
	USB-A to Micro-B Cable	N/A	1m USB-A to Micro-B Cable	1	1	1
					**Total**	**#VALUE!**
Michelson Interferometer*	HQ Camera Lens 6MM Wide Angle	Raspberry Pi	SC0124	25	1	25
	Ø12.7 mm Optical Post,L=100mm	Thorlab	TR100/M	7.14	6	42.84
	Ø12.7 mm Post Holder	Thorlab	PH75/M	10.29	6	61.74
	Ø1" Round Protected Aluminum Mirror, 3.2 mm Thick	Thorlab	ME1-G01	36.67	2	73.34
	Kinematic Mirror Mount for Ø1" Optics	Thorlab	KM100	44.78	3	134.34
	Ø1" 50:50 UVFS Plate Beamsplitter	Thorlab	BSW10	122.03	1	122.03
	USB WebCAM, Full HD 1080p 30FPS	Nordic	60074128	30	1	30
	0.8 mW Self-Contained (632.8 nm) HeNe Laser†	Thorlab	HNLS008L-EC	1337	1	1337
					**Total**	**1826.29**

‡ This laser has an estimated coherence length of 9 mm according to manufacturer specifications. A laser with a coherence length of 1 m or more would be preferable.
* The Michelson Interferometer is not essential for the LIL setup, and is used only to monitor the mechanical stability.
† The HeNe laser could be replaced with a high coherence length diode laser module at a cost of < USD 100, e.g. Integraf Holography Diode Laser (#DL-4B), USD 39
